# A Generic Model to Simulate Air-Borne Diseases as a Function of Crop Architecture

**DOI:** 10.1371/journal.pone.0049406

**Published:** 2012-11-30

**Authors:** Pierre Casadebaig, Gauthier Quesnel, Michel Langlais, Robert Faivre

**Affiliations:** 1 Unité de Biométrie et Intelligence Artificielle UPR 875, Institut National de la Recherche Agronomique, Centre de Recherches de Toulouse, Castanet-Tolosan, France; 2 Institut de Mathematiques de Bordeaux, Université de Bordeaux, Bordeaux, France; INSERM & Universite Pierre et Marie Curie, France

## Abstract

In a context of pesticide use reduction, alternatives to chemical-based crop protection strategies are needed to control diseases. Crop and plant architectures can be viewed as levers to control disease outbreaks by affecting microclimate within the canopy or pathogen transmission between plants. Modeling and simulation is a key approach to help analyze the behaviour of such systems where direct observations are difficult and tedious. Modeling permits the joining of concepts from ecophysiology and epidemiology to define structures and functions generic enough to describe a wide range of epidemiological dynamics. Additionally, this conception should minimize computing time by both limiting the complexity and setting an efficient software implementation. In this paper, our aim was to present a model that suited these constraints so it could first be used as a research and teaching tool to promote discussions about epidemic management in cropping systems. The system was modelled as a combination of individual hosts (population of plants or organs) and infectious agents (pathogens) whose contacts are restricted through a network of connections. The system dynamics were described at an individual scale. Additional attention was given to the identification of generic properties of host-pathogen systems to widen the model's applicability domain. Two specific pathosystems with contrasted crop architectures were considered: ascochyta blight on pea (homogeneously layered canopy) and potato late blight (lattice of individualized plants). The model behavior was assessed by simulation and sensitivity analysis and these results were discussed against the model ability to discriminate between the defined types of epidemics. Crop traits related to disease avoidance resulting in a low exposure, a slow dispersal or a de-synchronization of plant and pathogen cycles were shown to strongly impact the disease severity at the crop scale.

## Introduction

In agriculture, growers have to compete with harmful organisms (animal pests, plant pathogens and weeds), collectively called pests [1]. These pests have different ways to injure plants [2] and cause crop losses (quantitative or qualitative damages), causing economic losses. Among pathogens, diseases related to fungi can severely impair plant physiology by lowering light interception or reducing photosynthetic rate.

Thus, yield increases in the last decades resulted essentially from genetic progress and massive use of pesticides. However, current practices in crop protection generate a set of issues: (1) economical, as the cost of chemical treatments lowers margins when crop prices or yields are low; (2) durability, as the use of pesticides causes strong environmental risks: some pathogen populations can adapt and gain resistance rapidly (around 4 years); (3) technical, as the integration of alternative methods of crop protection (genetic, chemical, physical, biological and cultural control) is hard to achieve in a farm and finally (4) scientific, as the joint management of plant and pathogen populations in time (crop rotations) and space (landscape structure, [3]) is still a research question (integrated avirulence management, [4], [5]).

In a context of pesticide use reduction, alternatives to chemical-based crop protection strategies are needed to control diseases. Before the pesticide era, the canopy structure - resulting from the plant architecture and crop management - was the main natural lever to try to control disease development in cultivated fields. An update of this expertise is proposed through a research project focused on four architecturally contrasted systems and their main associated diseases: pisum-aschochyta blight, potato-late blight, vineyard-powdery mildew and yam-anthracnose systems. The first two systems were choosen as case studies for this paper. Knowledge in epidemiology tends to be very specific to the studied system. Modeling can help identify and share similarities between systems to unravel crop and pathogen interactions on a wider range of systems. Later, as a more operational step, modeling could be used to quantify how the canopy structure affects disease outbreaks and to identify architectural traits or cropping practices that lower disease severity. Provided that the model embeds genetic information (genetic basis of phenotypic parameters) it may later become a tool to design adapted genotypes [6], [7].

This scientific and operational context set several constraints on the model development. The main objective of this work was to cross the point of view of ecophysiologists and pathologists on the canopy function and structure to design a model with a generic scope, i.e. based on a single representation for different pathosystems, to explain contrasted dynamics of epidemics. Secondly, as the analysis to understand and use the model would be heavily based on simulation, the model design should minimize computing time by both limiting the complexity and setting an efficient software implementation.

Our aim in this paper was to present a model that suited these constraints so it can first be used as a research and teaching tool to promote discussions about the control of diseases.

We first present the conceptual design phase (*Analysis* section) that defines constraints for the following phase of model design and implementation (*Results* section). Model evaluation is presented in the *Discussion* section before presenting conclusions and perspectives.

## Analysis

Essentially, a crop-pathogen system can be viewed as the juxtaposition of individual hosts (plants) and infectious agents (pathogens) whose contacts are restricted through dispersal network; these three elements evolving under a fluctuating climate. By adopting a shared point of view on the crop and the pathogen, this dispersal network is defined by both the crop structure and the pathogen's dispersal mode. The properties of the generic objects defining the system are here detailed and are the conceptual basis of the model. This conceptual basis merges basic concepts from epidemiology, crop modeling and graph theory. The structure and dynamics of the formal model are presented in the *Results* section.

### Merging concepts from basic epidemiology, crop modeling and graph theory

#### Brief history of epidemic models in phytopathology

When observing the evolution of disease severity at the field scale, the crop leaf area is usually logistically destructed by the pathogen. As reviewed by Hau [8], two main approaches assuming different levels of complexity were investigated in the bibliography to model this observation: (1) mechanistic detailed models dealing with small-scale processes to understand the functioning of the whole system and (2) analytical models, based mostly on differential equations, with a limited number of processes and parameters [9]. The detailed approach tends to be very specific of the system (crop, pathogen species). An important feature of our approach is to build a model suitable for different systems, so we focused on the latter approach, based on simpler dynamics.

In the first models from Vanderplank [9] the evolution of disease severity (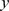) can be described by the logistic growth rate 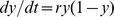 as a function of time (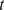) and an infection rate (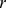). Such simple growth functions can be used to describe epidemics but not to explain the underlying biological processes [8]. Customized models could be built from this starting point to include biological processes such as the partitioning of the diseased individuals into different compartments (healthy, latent, infectious and removed) [10], namely SEIR models in human epidemiology. The interaction between host and pathogen growth could also be added to simpler models, as initiated by Jeger [11]. The main hypothesis underneath this model was the potential equilibrium state between host and pathogen populations. Such model design could extend to the integration of different organization scales still without using explicitly spatialized variables [12].

If further complexity is added in theoretical models, representing a generic behavior of a pathogen class (fungi) is trickier and less pertinent. Consequently, the computational study of the model is also more complicated. In analytical models, parameters such as 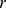 in the logistic growth contains information about the contacts underlying the disease transmission process. In this case, random contacts are assumed in the population (random mixing network). Considering non-mobile hosts, the number of contacts that each individual has is considerably smaller than the population size and, in such circumstances, random mixing does not occur [13]. It is thus important to model the contact network that depends not only on the type of pathogen's dispersion (wind, splashing, …) but also on the host's spatial arrangement.

#### Representing the canopy structure with a variable scale object

Plant epidemiology [14] and crop modeling [15], [16] are very active research topics but approaches that link these two domains are less frequent [2], [17], [18]. As the case studies for these models range from improving scientific knowledge to engineering, finding an appropriate coupling scale is difficult. For example, the effect of plant architecture on disease dispersal and severity was modeled by coupling a structural plant model (3D “virtual plant”) and experimental disease inoculations [19]. This organ-scale model would not be the most appropriate basis for adding dynamics of host or pathogen growth that would fit different crops, because of the overwhelming parameters fit needed. In our case study, we will use modelling concepts from crop models at a broad scale to allow an easier setup of generic features.

We assume that an approximation of the geometry rather than a fully detailed 3D plant structure is an acceptable simplification when considering the crop at an integrative level. When dealing with processes at crop scale, such as epidemic development, the underlying complexity could partly be discarded. If complexity is ignored, the model is able to describe the epidemic (using logistic growth for example) but significant interactions between elements of the system will not be highlighted.

We have chosen to keep sub-crop scale objects (plants or organs) but discard their geometric attributes (size, shape, spatial position and orientation). The canopy was represented by an object that was simply named *functional unit*, the scale of this object was relative to the epidemic type. It was also more convenient to describe processes at the level where they are better known for each system. For example, in dense canopy, where epidemics tend to ascend from lower crop layers [20], individual plants are less pertinent than a representation of a mean plant divided in stem segments (phytomers). In patchy epidemics, individual plants get infected and disperse infectious agents in their local neighbourhood. These two epidemic archetypes were taken as a case study. Thus, functional units can represent either a mean plant, individual plants or a sub-part of a plant. The canopy architecture was characterized by a network of functional units. Dynamics of plant and pathogen growth were described on functional units, meaning that a homogeneous behavior of plant tissues was supposed within the unit.

#### Modeling spore dispersal by using a graph-theory perspective

Early epidemiological models (logistic growth or SEIR-derived) have a long and successful history but are based on population-wide random mixing [13]. In agronomic systems, where canopy results mainly from interaction between cropping practices and the environment, there is often a strong spatial structure of the host population (rows, trellis). In practice, each individual has a finite set of contacts that they can infect. An analogy with graph theory, with hosts as graph nodes and neighbourhood of infection as edges, seems an adapted approach to deal with these limited contacts [13], [21]. In our model, a graph, namely the network of connections between functional units, was used to represent the spore dispersal process.

Setting up an experiment to determine the real network of connection is tedious for spore-transmitted infections as relationships between individuals are not observable in field. Therefore, the representation of the real network results from an *a priori* point of view on both crop structure and dispersal mode rather than direct observation.

## Results

### Model overview

**Figure 1:**
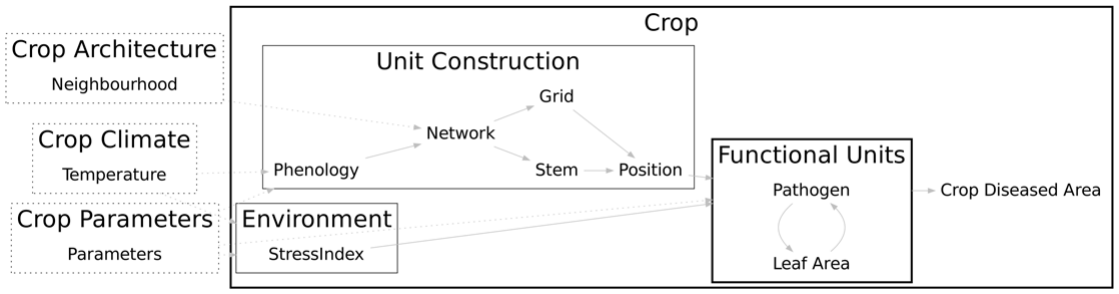
General model structure. This summary view represents a hierarchical organization of sub-models which describes objects and processes of the system. Model inputs are shown in dotted frames. Scales (crop and unit) are shown in bold lines. Sub-model *Unit Construction* acts as a controller for the dynamically created and individual based models *Functional Units*.

The epidemiological model consists of individual-based sub-models for pathogen and leaf area dynamics linked together by a network that represents the canopy architecture. Figure 1 shows these two scales, a global one (crop) and a local one (individual hosts referred to as functional units) which interplay by using common attributes. This conception allowed individual host dynamics to be affected by global crop properties that are in turn dependent of individual attributes. Figure 1 presents a static view of the model before the beginning of the simulation. As the simulation progresses, functional units are created and linked together according to the specifications of the connection network.

The canopy structure and development processes are detailed in the *Unit Construction* sub-model. The *Functional Units* model describes the dynamics of plant (leaf area) and pathogen (leaf area injured) growth at local scale as a set of ordinary differential equations. The environmental effect on pathogen growth is computed in the *Environment* sub-model.

The formal model is deterministic with a discrete time-step (daily). Model's variables are summarized in table 1. System input variables are climatic measurements (mean air temperature). Input parameters are defined in table 2.

**Table 1:** Summary table for variables.

symbol	name	unit	eq.
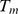	mean air temperature	°C	
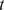	thermal time	°C 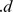	
	unit number (parameter in “grid” method)	-	
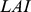	leaf area index	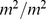	
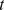	thermal age	°C 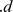	1, 2, 3, 5
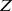	height	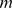	1
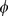	porosity	-	6
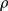	receptivity	-	5
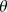	thermal stress	-	8
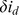	allo-deposition	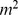	7
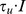	auto-deposition	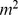	4
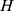	healthy area	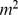	4
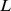	latent infected area	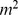	4
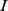	infectious area	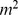	4
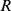	removed area	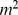	4
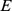	expanded area	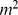	2
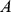	photosynthetically active area	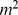	
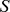	senescent area	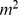	3

Horizontal lines separate crop attributes from unit specific attributes. Rate-based variables directly linked to state variables were not specified in the table.

**Table 2:** Summary table for parameters.

symbol	name	stem	grid	uncertainty	unit	eq.
*T*_b_	base temperature	0	4		°C	
*t_u_*	phyllochrons	70 ; 50	NA		°C .*d*	
*t_f_*	crop thermal time to flowering	1500	2000		°C .*d*	
*Z_max_*	potential height of unit	1	1		*m*	1
*k_z_*	increase of elongation rate	0.01	0.015		°C^−1^.*d* ^−1^	1
*t_z_*	unit / crop thermal time for 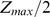	300	1000		°C.*d*	1
*E_max_*	potential area of unit	1	1		*m^2^*	2
*k_e_*	increase of expansion rate	0.01	0.015		°C^−1^.*d* ^−1^	2
*t_e_*	unit / crop thermal time for 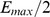	300	1000		°C.*d*	2
*k_s_*	increase of senescence rate	0.03	0.06		°C^−1^.*d* ^−1^	3
*t_s_*	unit / crop thermal time for 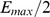	1000	2500	[70; 130] %	°C.*d*	3
*ip*	duration of infectious period	10	10	[1]; [15]	*d*	4
*lp*	duration of latency period	3	5	[1]; [15]	*d*	4
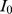	quantity of inoculation	0.01	0.01	[0.01; 0.3]	*m^2^*	
*D_i_*	day of disease initiation	50	50	[30; 140]	*d*	
*τ_n_*	scalar for between-unit infection rate	0.4	0.5	[0.05; 1]	-	4
*τ_u_*	scalar for within-unit infection rate	0.6	0.5	[0.05; 1]	-	4
*t_r_*	shape of receptivity function	500	500	[100; 800]	°C.*d*	5
*k_r_*	slope of receptivity function	0.05	0.01		°C^−1^.*d* ^−1^	5
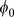	scalar for porosity level	0.8	0.9	[0.7; 1]	*m* ^−1^	6
*T_io_*	optimal temperature for infection	25	25	[10]; [35]	°C	8
*T_iw_*	shape parameter of infection response to temperature	2.2e^−3^	2.2e^−3^		-	8

Horizontal lines separate the parameters related to canopy, pathogen and environment. Columns “stem” and “grid” indicate the value of parameters for the specific use-cases. Column “uncertainty” indicates the range of selected parameters in sensitivity analysis (in % when the meaning of the parameter is different in both models).

### Canopy development (*Unit Construction* sub-model)

In plant models, time scale is frequently coupled with temperature as most physiological processes are temperature-dependent. The concept of thermal time [22] implies that time, as sensed by plants, elapses more rapidly at higher than at lower temperatures in a given range of temperatures [23] and is stopped at a base temperature. It is convenient as dates or rates expressed in thermal time are constant between environments. The main development stages for the crop (emergence, flowering, maturity) are expressed as a sum of degree-days (mean air temperature). As a consequence, calendar time (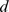, day) and thermal time (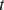, °C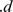) were defined as different mathematical notations.

In the *Unit Construction* model, the development process of the canopy was represented by the creation of functional units. This structural aspect is distinct from the functioning of each unit. Two pathosystems were taken as example to test the model genericity: (1) the pisum-aschochyta system [20], where the description of epidemics combines a local spore dispersal (splashing, stem-flow) and organ scale knowledge and (2) the potato late blight system that with long distance wind-dispersal between individualized plants [24]. Two corresponding methods, respectively named “stem” and “grid” are presented here to generate these contrasted canopy archetypes.

**Figure 2:**
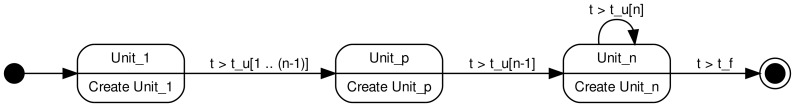
Hortel statechart for the “stem” method. Hortel statechart for semi-determinate development of plant structure. The conditions for building a new unit (i.e thermal time above a threshold) are specified in transition between states and represents phyllochrons (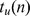) or crop flowering time (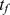).

**Figure 3:**
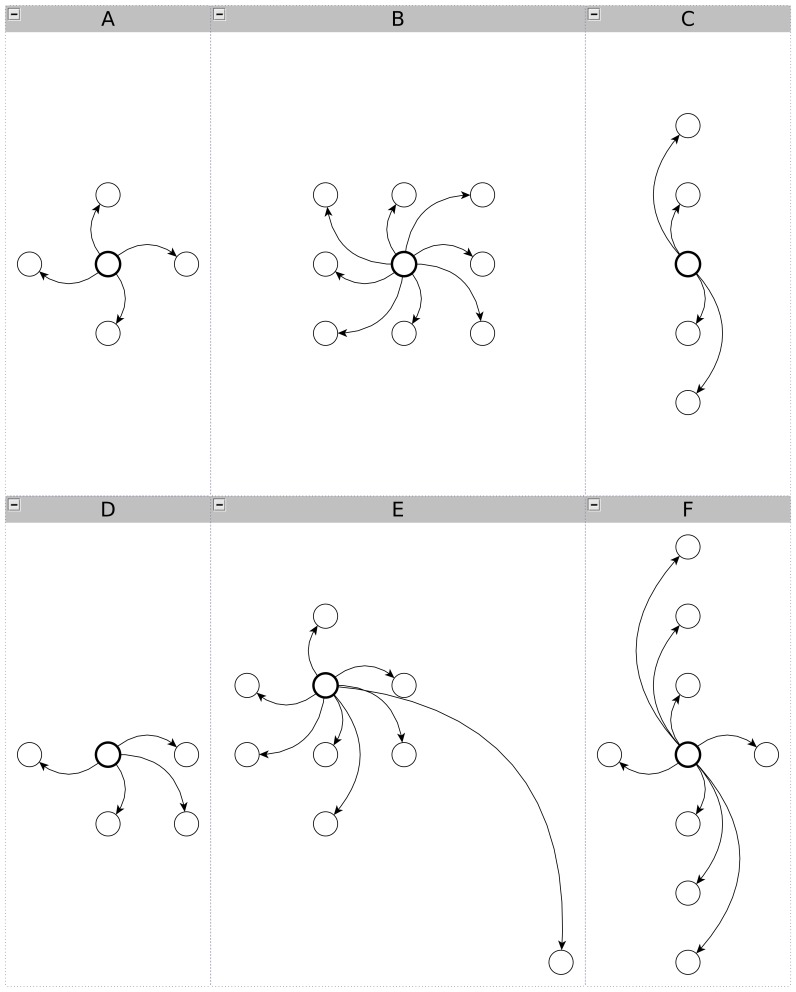
Individual neighbourhood for the grid method. Nodes are individual plants and edges are pathogen dispersal processes. Local dispersion graphs are defined from a individual unit point of view (bold circle) and are used to infer the global network of connections between all functional units at the crop scale.

***Stem method*** is used to simulate pathosystems presenting homogeneous canopies where epidemics follow a vertical dynamic. In this method, a single plant is represented as a succession of growing stem segments. The hypothesis of an homogeneous crop in the plot (mean plant) allowed aggregation at crop scale. The horizontal plot variability coming from soil, climate or primary inoculum repartition was not considered. In the model, functional units are created sequentially and recursively linked together to mimic stem growth. Thus, each functional unit represents a layer of organs having a similar physiological age over the whole canopy. The dynamic construction of the units was implemented with a finite state automaton (modelled with an Hortel statechart [25], a variant of the UML statechart [26]). In the statechart presented in figure 2, states correspond to units and the transitions between states are conditioned by thermal time accumulation. Different phyllochron values were defined to simulate preformed stem segments (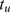 is a vector). The unit construction process stops at flowering time (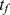), determined at crop level. This method also defines the relationship between units: each unit is connected to its upper neighbour (linear graph).
***Grid method*** is used to simulate patchy epidemics, with each unit representing an individual plant. In this case, all units are created simultaneously at the start of the simulation and are linked according to a network of connection. This global network is build from an *a priori* on the neighbourhood of individual units about both the pathogen dispersal process and the crop structure. This neighbourhood, relatively to individual units, is defined as a deterministic parameter (graph). Examples of possible individual neighbourhoods are presented in figure 3. The strength of the dispersal process was related to the number of plants considered in theses graphs (i.e graphs A *vs* B and D *vs* E). Constraints on range or direction of the dispersal process are represented by defining asymmetric graphs (D, E, F). For example, graph E is used to simulate long-range dispersal events occurring at a low frequency. Graphs C and F are defined to represent row-structured crops, with a weak inter-row connection for graph F. At the crop scale, this theoretical network of unit connections is parameterized by the adjacency matrix of the corresponding graph. The outward edges of each node are weighted to adjust dispersal rate in relation to the leaf area development of the emitting unit (porosity attribute, cf. eq. 6).

### Individual dynamics for plant and pathogen growth (*Functional Units* sub-model)

The dynamic of functional units, whether they represent plants or organs, was defined by two interacting equation systems. The first one is related to healthy plant functioning and the second depicts disease development on leaf tissues. These two systems include terms that depend on state variables from linked functional units, allowing to model disease development at the canopy scale.

Functional units were mainly described by leaf area because it is a driving variable for light interception (linked to crop productivity) and a physical support for pathogen development. The units also included height and porosity attributes. As in SEIR family models [4], [27], total leaf area for each functional unit is divided into distinct compartments. From the plant point of view, we defined expanded (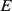), senescent (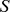) and photosynthetically active (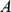) leaf area. For the pathogen, the healthy (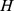), infected (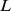, Latent), infectious (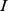) and removed (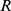) compartments. The *removed* class combined leaf area destruction caused by natural senescence and by disease development. If there is no infection, all the leaf area is thus removed through natural senescence. At each time-step, the identity 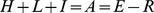 holds to maintain leaf area conservation (as for biomass or energy conservation).

#### Dynamics of plant elongation, leaf expansion and senescence

Stem height elongation (eq. 1), leaf area expansion (eq. 2) and leaf senescence (eq. 3) rates were modeled with a symmetric logistic equation driven by thermal time 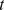 and 3 parameters. This formalism is frequently used at the crop scale in ecophysiology. It was previously adapted at organ-scale [28]. This approach is similar to ours when considering an analogy between organs and functional units.
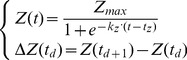
(1)

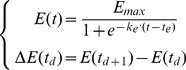
(2)

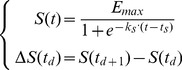
(3)

*Emax;Zmax* the value of asymptote, representing the potential of the elongation (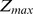), expansion or senescence (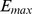) process whether a whole plant (total leaf area, plant height) or an organ (phytomer area, internode length) is considered. These parameters are measurable on plants growing in healthy conditions and without environmental stress.
*te; ts; tz* the value of the abscissa at the inflection point, representing thermal-time at half the value of 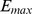 or 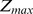. Their value was estimated through optimization. We stated that 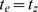, meaning that elongation is synchronized with expansion.
*ke; ks; kz* the value of the slope at the inflection point. This value is supposed to be constant among functional units (different between processes) and was optimized.

This model relies on two hypotheses: (1) the disease development does not affect the plant growth rate (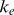) nor the potential size (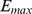), (2) there is no specific feedback of the disease on natural leaf senescence (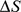 is disease-independent).

#### Dynamic of leaf area in disease condition

The diffusion of pathogenic agents (i.e. spores) was not modelled explicitly and is approximated by rates of conversion between the different leaf area compartments. The aim of the pathogen model (eq. 4) was to merge a compartmental model of leaf injury and the host plant growth. This model was adapted from previously developed model on the powdery mildew - vineyard system [29]. In our case, two processes were refined: (1) healthy area was dependent of host growth and was simulated with a basic crop model and (2) both pathogen-induced and natural senescence (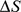) contributed to the removal of leaf area from the system. It was also assumed that the natural senescence process removed leaf area independently from its health status (infected or infectious). It was a way to model disease resistance via avoidance, as leaf area can be removed before it became infectious.
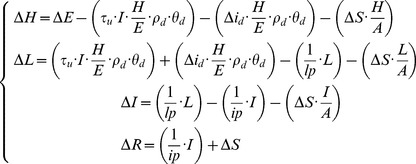
(4)


*Disease initiation*. In the model, the primary infection event occurs at a pre-defined time (at day 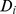) and a fraction of healthy area (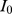) is converted into the infected state. Depending on the epidemic archetype, either all units (for the “Stem” model) or a fixed proportion of the existing units at the time of the infection (“Grid” model) are affected. *Disease's development*. The dynamic for healthy area (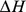) is given by the host growth (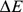) *minus* the area infected from a local source *minus* the area infected from the neighbourhood *minus* the area that became senescent. In consequence, two parameters acting as rates of infection drive the disease progression in the unit (auto-deposition, 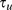) and between neighbouring units (allo-deposition, 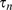). The other terms in equation 4 are either regulation function (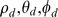) or normalizing terms (area in the compartment divided by total area). The disease development is also a function of a latency period (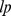) and an infectious period (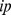). For computing simplicity, these periods are not simulated as a time-lag model, meaning that a fraction of infected area becomes infectious from day one after infection (with a rate of 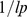).

*Disease regulation by crop properties*. Receptivity, namely the level of ontogenic resistance of the plant tissues, affects local and distant infection rates. Equation 5 presents an example of decreasing ontogenic resistance with the unit aging and showing an increase in the rate of infection on the onset of leaf senescence (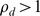). Ontogenic resistance was modeled as a pathosystem-specific function [30] as tissues could get less resistant to infection with aging (pisum-aschochyta [31], potato late blight [32]) or, conversely, could become nearly immune in older organs (vitis-powdery mildew [33]). Biotrophic or necrotrophic behaviours were partly reproduced by this function.

The attribute 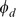 of unit porosity integrates the architectural characteristics (e.g leaf area orientation, leaf surface properties) that are assumed to impact disease transmission and which are not explicitly modeled. Porosity is approximated (Eq. 6) by leaf area density (area/height) weighted by parameter 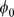. This regulation through porosity impacts the rate of infection from neighbouring units.
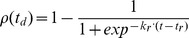
(5)

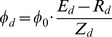
(6)


#### Canopy architecture impact on disease transmission

Predicting the population dynamics from the behaviour of individuals is a challenge when modeling epidemics [34]. To face this challenge, we formulated two hypotheses: (1) the individual-based compartment models (Eq. 4 and 7) are functions of crop-scale attributes, to make individual dynamics sensitive to global conditions and (2) the number of connections between units depends on the pathogen dispersal mode. The outgoing infection from a unit 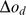 is modeled as multiplicative effect of infected area and unit porosity (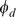). For each unit, the incoming infection (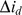) is modeled as the sum of neighbouring emissions (cf. fig. 3.). Input and output disease flows are not synchronized: emissions are computed at the next time-step (the day after) in the target units.
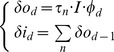
(7)


### Impact of environmental stress on plant or pathogen dynamics (*Environment* sub-model)

The *Crop Climate* model aimed to compute environmental impacts on pathogen development. At this step, only the temperature effect on disease development is modeled (Eq. 8). This factor was introduced as a multiplicative term that modulates disease transmission rates within and between units (Eq. 4). This bell-shape function depends on the crop mean temperature (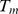) and two parameters: the optimum temperature for pathogen growth (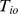) and the width of the response at this optimum temperature (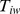). The parameter 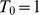 was defined as a normalizing term to allow dimensional consistency.
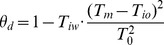
(8)


### Software implementation and data analysis

#### Software implementation

The model development and simulations rely on the VLE software platform (Virtual Laboratory Environment) [35] and the INRA RECORD platform [36]. This platform implements a mathematical specification (DEVS, Discrete EVent system Specification [37]) that was originally developed for research in modeling and simulation independently of the domain of application. The main feature of the VLE platform is that it proposes software sub-formalisms between the abstract DEVS specifications and common mathematical formalisms in environmental sciences (e.g ODE, Hortel statechart, cellular automatons). Consequently, most of the formal model was implemented by using these extensions rather than manipulating underlying DEVS formalism and making it possible to integrate heterogeneous formalisms in the same model. *Dynamic creation of model structure*. Following the class/object paradigm, the *Functional units* model is defined as a class model whose instantiation depends on the model graph structure. Depending on the pathosystem, instantiation is static (at plant emergence) or dynamic during simulation (following the plant development); these new units interact according to the specification of the connection network. This allowed dissociating the biological representation from its consequence on the graph and simulation.

#### Data analysis

The sensitivity analysis and data visualization was performed with R core software [38] with R packages *sensitivity* and *ggplot2* [39]. Global sensitivity analysis aims at explaining the variability of model outputs with respect to the input parameter uncertainties. It is based on intensive use of computer simulations. After setting an experimental design of parameter values, model outputs are evaluated for each parameter set and sensitivity indices are computed. Various methods of global sensitivity analysis exist [40]. To avoid the 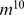 model evaluations of a full factorial design (10 parameters with each m modalities), we used the extended-FAST method [41] (size 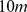, with 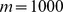) to perform the sensitivity analysis. It allows to evaluate a first-order sensitivity index (influence of only one parameter on the output) and a total sensitivity index (influence of one parameter alone and in interaction with the others parameters). These two indices correspond, in the classical variance analysis, to the principal effect and the total (principal+interactions) effect.

## Discussion

### Qualitative model evaluation

A strong hypothesis is that the model genericity is fully supported by the parameter set (cf. table 2) and the individual graph generating the global connection network (cf. figures 2 and 3). This hypothesis was tested here by running the model with two sets of parameters. The first set of parameter values characterizes a vertical epidemic in a homogeneously layered canopy whereas the second represents a spatial epidemic in a field (lattice of individual plants). These parameter sets will be later referred to as vertical or spatial epidemics. In our approach, the model has been built from a conceptual point of view to get gradually closer to the biological reality. In this sense, the parameter estimation on real data was not our priority in the development of the model. Nevertheless, some parameters are quite easy to estimate, especially those related to phenology as they are well studied for a number of crops (e.g. in pea [42], [43], potato [44], [45]). In the present work, parameters were not estimated with a numerical optimization method, but were defined so that the epidemic dynamics are meaningful according to the pathologists' knowledge.

The first step concerning the model evaluation was to reproduce real behaviours, those that pathologists observe in field conditions. The output variable used to evaluate the model and perform a sensitivity analysis is the proportion of leaf area removed by the sole effect of disease (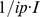), integrated over time (sowing to harvest) and space (units).

#### Ascochyta blight on pea

**Figure 4:**
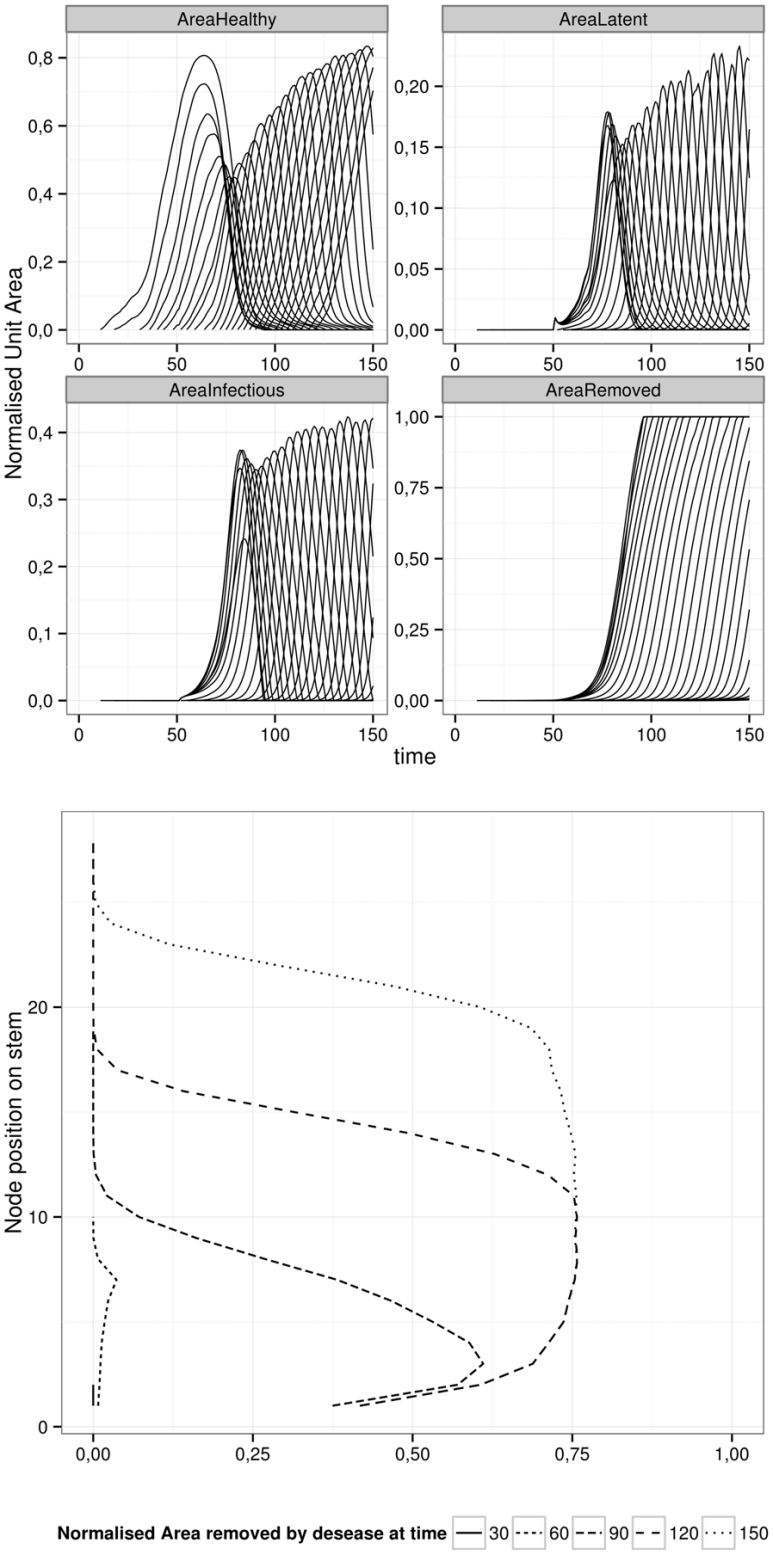
Vertical epidemics: disease development in a homogeneously layered canopy. The four upper graphs show individual dynamics of functional units, with each graph corresponding to a state of infection. The output variables are relative to the total leaf area in the unit. The lower graph presents the evolution of “infection profiles”, namely the severity of the disease in relation to the height (stem nodes) in the crop.

The stake was to reproduce “infection profiles”, i.e. a varying disease score upon the canopy height, caused by the development of ascochyta blight on pea fields [20]. This observed behaviour is assumed to be a consequence of two processes: a frequent disease initiation on lower leaves (older and more receptive) and a local dispersion to upper nodes [31]. The bell shape of the disease profiles is interpreted as a gradient of age-based (ontogenic) resistance, as upper and younger leaves are more resistant to infection and lower leaves could become senescent before being fully infected. The model outputs are presented in figure 4. They display a classical behaviour as the disease starts developing firstly on lower nodes, moving upwards with time (figure 4, lower graph) [46]. Even if individual unit dynamics are rarely observed in field, the upper section of figure 4 indicates that after the beginning of infection (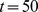), the system was perturbed to time about 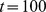 before returning to a healthy state. This result is interpreted by the combination of two effects: a high host growth rate (caused by a favorable oceanic climate) and a weak disease transmission rate between plant nodes (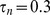). Refining the model prediction at this scale will require additional experiments on controlled conditions (e.g. on leaf receptivity, [31]).

#### Potato late blight

**Figure 5:**
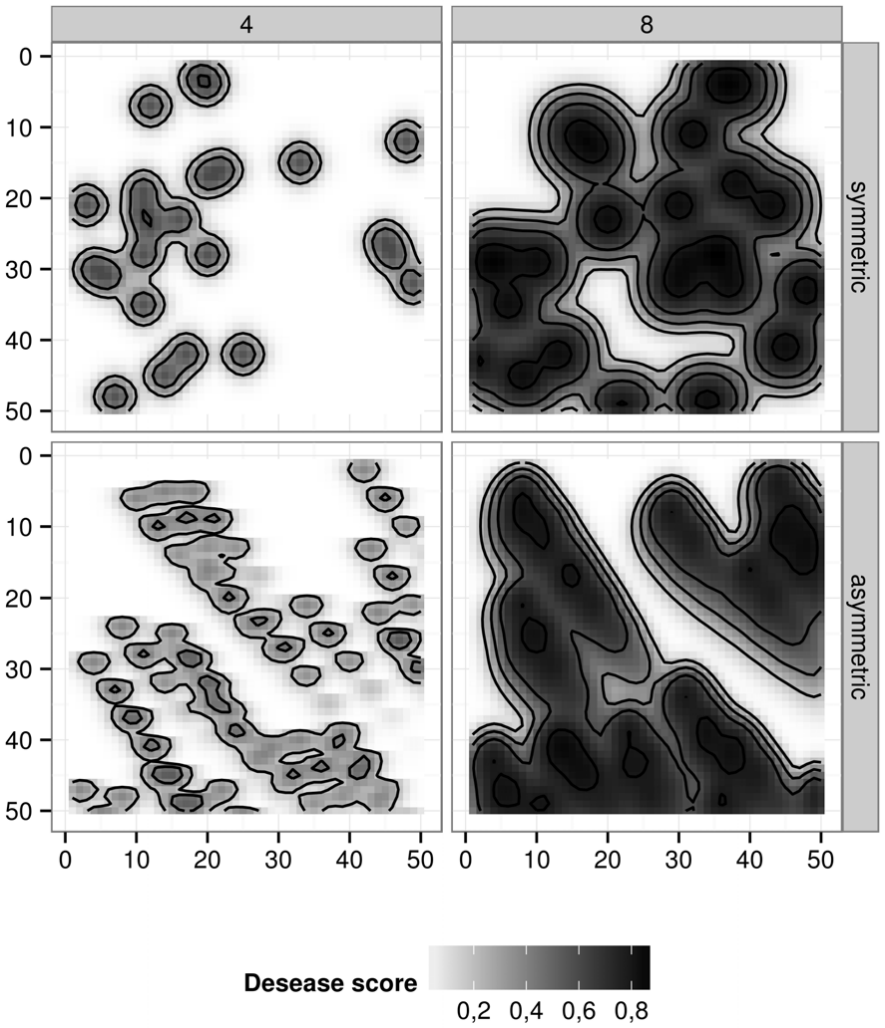
Spatial epidemics: disease development in a lattice of individualised plants. The four maps show the disease severity (grey scale) on grids of 50×50 plants at the end of simulation. Disease initiation was done at random on 1 percent of total cell number for both cases. These maps differed in the parameterization of the connection network between plants (cf. figure 3): upper maps resulted from symmetrical graphs (i.e. graphs A and B in figure 3), lower maps resulted from asymmetrical graphs (D and E); right and left maps resulted from a variation in the number of plants considered for local dispersion (4 and 8).

In this case, the model parameters intend to represent a spatial epidemic, where random primo-infection generates primary foci which in turn infect plants in their neighbourhood [24]. It is not clear how to infer the connection network from the real system. On one hand, observation of patches of infection means a short-range diffusion process (new infections are more likely to occur near a previously infected plant). On the other hand, a long-range dispersal is often observed [47]. Thus, the simulations showed in figure 5 aim at exploring the sensitivity of the epidemic process to changes in size or range in the dispersal neighbourhood. Four examples were chosen from graphs in figure 3. The asymmetry of some graphs, used as connection network, provide interesting heterogeneity at the canopy scale. This parameterization could represent epidemics in heterogeneous fields (e.g. prevailing winds, asynchronous plant emergence). These results illustrate the plasticity we achieved in simulation rather than a formal sensitivity analysis on this connection network.

With our model, a quantitative proportion of injured leaf area is used as output data, as pest injuries are usually observed on quantitative scales. Observations to discriminate between pathogen-induced or senescence-induced removal of leaf area is a very difficult task, if observation is not performed continuously. This modeling approach permits to estimate the respective proportion of leaf area removal caused by these two processes. Protocols for characterization of pest injuries in the field [48] should be addressed in order to evaluate the predictive quality of our modeling.

### Sensitivity analysis

We have seen in the former section that it was possible to reproduce observed real behaviors using specific sets of parameter values. Sensitivity analysis can be useful to determine which parameters are the most influential on the variability of the model output, which are those having no influence (precise knowledge on these parameters may not be not necessary), which are those interacting together. It can help the modeler to check if the model respects the *a priori* knowledge on relative effect of parameters and their interactions. This analysis can also help set research priorities. For example, to help decide which experiments are needed to improve the precision on the estimation of some parameters or to identify regions of the parameter space with a high (or low) model output which can be useful on a prospective point of view.

**Figure 6:**
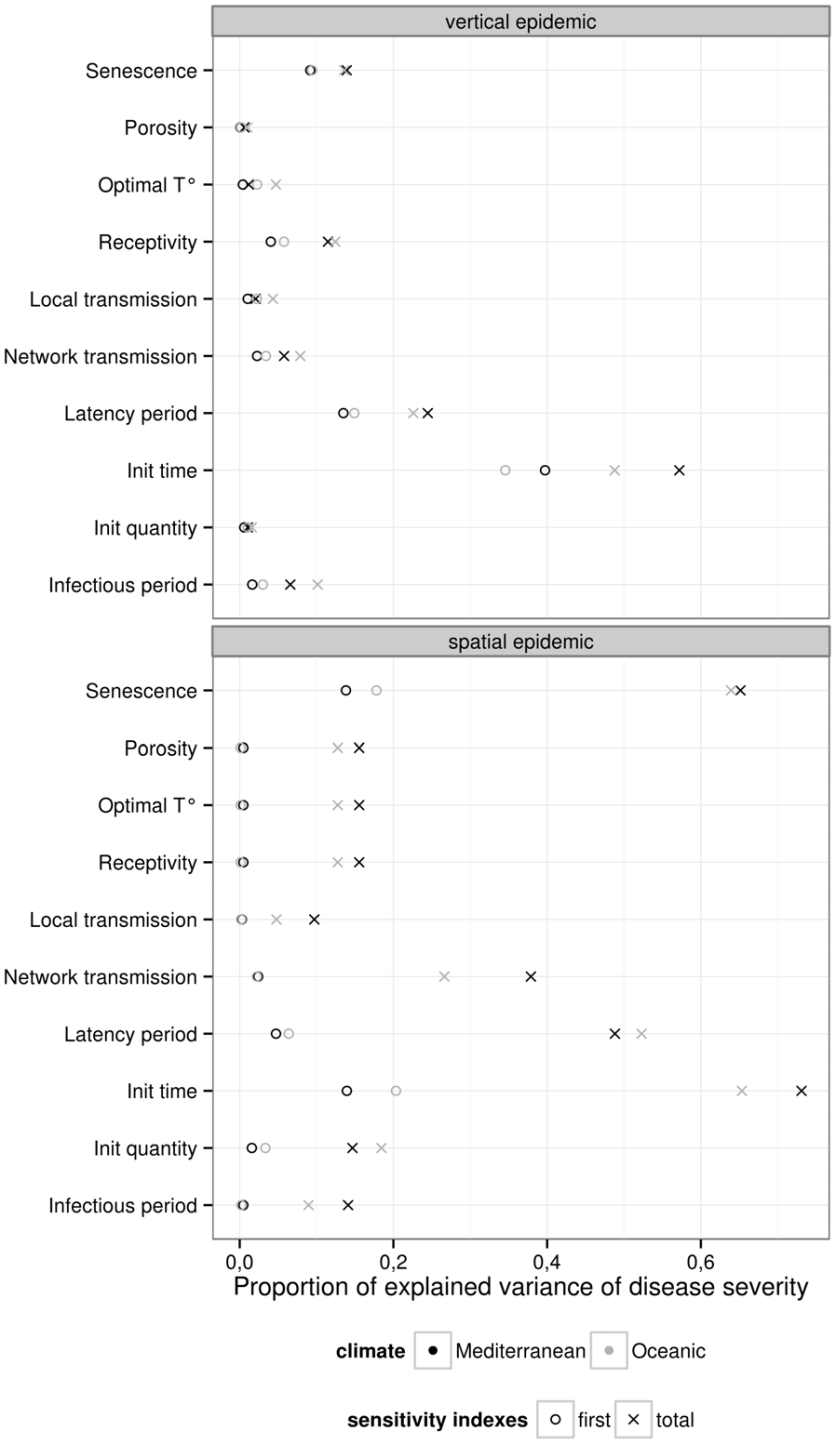
Sensitivity analysis. Impact of 10 parameters from table 2 on disease severity for the two pathosystems: top, ascochyta blight on pea, and bottom, potato late blight. Main effect (circle) and total effect (cross) for each parameters are estimated with Extended FAST method [41] on two contrasted climatic conditions: Mediterranean (black) and oceanic (grey).

A global sensitivity analysis of the model was conducted to further apprehend its behavior (cf. figure 6). The impact of 10 parameters on disease severity at the crop scale was assessed for the two pathosystems (ascochyta blight on pea and potato late blight) on two contrasted climatic environments (Oceanic - Rennes, Western France and Mediterranean - Montpellier, South of France). The range of variation for the input parameters was adjusted to keep a biological meaning. We assumed a uniform distribution within this range (figure 6, column “uncertainty”). The Extended-FAST method [41] was used to explore parameter space and compute sensitivity indices.

Whatever the model, the three most influential parameters on disease severity are the disease initiation day, the latency period and the onset of senescence. All three parameters are time-related. This analysis points out that avoidance strategy strongly explains disease severity: whether this avoidance came from a low exposure (late inoculum arrival), a slow disease dispersal (long latency period) or a de-synchronisation of plant and pathogen cycle (early plant senescence). The high impact of the disease initiation day parameter on the severity suggests that the control of primary inoculum production is a key point for crop protection. This point should be addressed in connection with management strategies, i.e. interaction between tillage and crop sequence to manage infected stubble, [49].

As expected, estimated sensitivity indices highlight the strong impacts of both the network transmission and senescence parameters. The impact of senescence on severity is higher in spatial than in vertical epidemics. This is interpreted as the consequence of the synchronisation of host development in the spatial model. Interestingly, all parameters interacted more tightly with each other in spatial epidemics (larger total index).

Both models are sensitive to climate (temperature) and show contrasted response. In Mediterranean climate, the disease severity is globally more sensitive variation of model parameters, for the two types of epidemics. In the warmer climate, disease initiations concomitant with a senescent crop (resulting from a hastened crop development) appeared to be more frequent and thus a higher epidemic variance was observed. For the spatial epidemic case, the impact of host ontologic resistance (receptivity) and optimal temperature for pathogen development shifted from none to significant with the change in environmental conditions. This indicates that the model is able to reproduce interesting host
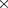
pathogen
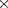
environment interactions and could be useful to better understand some mechanisms of plant epidemics.

### Conclusions

As stated before, the developed model lies in the research domain. It needs more modeling iterations with phytopathologists to be able to produce outputs that can be used for agricultural applications. Nevertheless, the modeling process raises interesting questions that could be scrutinised with experimental approaches: the disease impact on plant senescence, previously viewed as an additional process to the model, was finally represented as a simple competition for leaf area. Simulation plays a great role to help grasping the functioning of agricultural systems where direct observations are difficult and tedious, providing a sound balance between real and virtual experiments.

#### About conceptual development

A consequent part of the model adaptation to specific systems was achieved through the structure of unit network. As its impact cannot be directly evaluated through classical sensitivity analysis, additional virtual experiments would be necessary to develop an intuitive understanding of network-based epidemics and the effect of network structure [13]. Thereby, to simulate systems with a high number of units and a nearly-complete connection graph (e.g. vineyards) will be highly demanding in computing time: statistics on connection network on real pathosystems would help reduce this uncertainty. A first step, to improve the present model, is to focus on the climatic environment at the local scale (microclimate or phylloclimate [50]) as this local heterogeneity could probably explain finer host
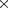
pathogen
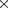
environment interactions. The modular model conception makes this improvement easy to take into account. Additionally, environmental stresses are most probably not sensed independently by plant (or organs). An interesting model combining the action of temperature and wetness on pathogen response, proposed by Duthie [51], could be considered to integrate the joint effect of stresses for a range of foliar pathogens. Cropping practices (e.g. pruning, lodging) that dramatically affect the crop structure are not yet included in the present model and should improve its relevance for practical applications.

#### About practical development

The modeling of two types of epidemics within one single model structure was a first step demonstrating that the defined objects (functional units and a network of connection) had sufficient flexibility to mimic different crop/pathogen systems. Because we focused on comparative epidemiology rather than on quantitative evaluation, operational conclusions are still out of the scope of the model, and are therefore not presented in this paper. Very few studies have integrated canopy architecture and functioning as a way to control disease [52], [53]. Among these studies, the crop-pathogen system is mainly modeled with functional-structural plant framework (FSPM) including 3D representation of the canopy. Using a coarser-grained and less computationally intensive model makes uncertainties (climate variability and change, diverse production systems) easier to analyse within a simulation scheme. Biological complexity is thus easier to integrate and assess [54].

Integrative biology can be seen not only as integration between scales but also between the different points of view (agronomists, pathologists and mathematicians). This work also highlighted the role of software simulation platforms as tools to integrate the knowledge coming from these different fields [55].
